# Surface Roughness and Biocompatibility of Polycaprolactone Bone Scaffolds: An Energy-Density-Guided Parameter Optimization for Selective Laser Sintering

**DOI:** 10.3389/fbioe.2022.888267

**Published:** 2022-07-11

**Authors:** Jian Han, Zehua Li, Yuxuan Sun, Fajun Cheng, Lei Zhu, Yaoyao Zhang, Zirui Zhang, Jinzhe Wu, Junfeng Wang

**Affiliations:** ^1^ High Magnetic Field Laboratory, CAS Key Laboratory of High Magnetic Field and Ion Beam Physical Biology, Hefei Institutes of Physical Science, Chinese Academy of Sciences, Hefei, China; ^2^ University of Science and Technology of China, Hefei, China; ^3^ School of Basic Medical Sciences, Anhui Medical University, Hefei, China; ^4^ School of Electronic Engineering and Intelligent Manufacturing, Anqing Normal University, Anqing, China; ^5^ School of Electronic Engineering, Naval University of Engineering, Wuhan, China; ^6^ Institutes of Physical Science and Information Technology, Anhui University, Hefei, China

**Keywords:** selective laser sintering, surface roughness, biocompatibility, energy density model, polycaprolactone

## Abstract

Three-dimensional porous polycaprolactone (PCL) bone scaffolds prepared by selective laser sintering (SLS) have demonstrated great potential in the repair of non-load-bearing bone defects. The microgeometry and surface roughness of PCL scaffolds during the SLS process may change the biocompatibility and bioactivity of the scaffolds. However, in addition to the widely concerned mechanical properties and structural accuracy of scaffolds, there is still a lack of systematic research on how SLS process parameters affect the surface roughness of PCL scaffolds and the relationship between roughness and biocompatibility of scaffolds. In this study, we use the energy density model (EDM) combined with the thermodynamic properties of PCL powder to calculate the energy density range (Ed_1_–Ed_3_) suitable for PCL sintering. Five PCL scaffolds with different laser powers and scanning speeds were prepared; their dimensional accuracy, mechanical strength, and surface properties were comprehensively evaluated, and the bioactivities were compared through the attachment and proliferation of MC3T3-E1 cells on the scaffolds. It was found that the high energy density (Ed_3_) reduced the shape fidelity related to pore size and porosity, and the dense and smooth surface of the scaffolds showed poor cytocompatibility, while the low energy density (Ed_1_) resulted in weak mechanical properties, but the rough surface caused by incomplete sintered PCL particles facilitated the cell adhesion and proliferation. Therefore, the surface roughness and related biocompatibility of PCL bone scaffolds should be considered in energy-density-guided SLS parameter optimization.

## 1 Introduction

Polycaprolactone (PCL) has attracted extensive attention owing to its good biodegradability and biocompatibility and has been approved by the U.S. Food and Drug Administration (FDA) as an implantable biomedical material for tissue engineering (TE) (Yeong et al., 2010; Munir and Callanan, 2018; Farazin et al., 2021; Navaei et al., 2021). Due to its relatively poor mechanical properties, PCL matrix bone scaffolds were usually utilized to repair the non-load-bearing regions, such as cartilage (Hajiali et al., 2018). The biocompatibility and repair ability of bone scaffolds are closely related to their surface properties. Many researchers are focusing on the chemical and biological activities of the scaffold through surface coating; the physical properties of the scaffold surface, including the surface microgeometry and roughness, can also significantly alter the biocompatibility and repair ability of the scaffold (Bachle and Kohal, 2004; Kim et al., 2004; Czelusniak and Amorim, 2020). For example, Jeon et al. (2014) have manufactured PCL scaffolds and then modified the scaffold surface *via* oxygen plasma treatment. Their results indicated that appropriate roughness induces favorable cell responses. Borsari et al. (2005) utilized two innovative vacuum plasma sprayed (VPS) coating techniques, with coated hydroxyapatite (HA) on Ti_6_Al_4_V scaffolds. The results have shown that the surface morphology and the HA coating strongly affected cell behavior. Mustafa et al. (2001) varied the surface roughness of the titanium implant material and examined the effect of cellular attachment, proliferation, and differentiation. There are a lot of reports which said that the selective laser sintering (SLS) technique can fabricate PCL scaffolds with a rough surface.

As a three-dimensional (3D) printing technology, SLS uses CO_2_ laser to sinter polymer thin layers or their composite powders to form solid 3D objects. It is self-supporting, has high precision, is customizable, and is particularly suitable for making porous scaffolds (Du et al., 2017). Different from the traditional method of surface reprocessing on the prefabricated scaffold, SLS can effectively control the surface microgeometry while 3D printing the scaffold. By adjusting the process parameters, including laser power, scanning speed, layer thickness, incubation space, and powder bed temperature, SLS provides users with good control over the surface roughness of the scaffold (Beal et al., 2009; Duan and Wang, 2011; Sachdeva et al., 2013). PCL is a semicrystalline synthetic polymer with a low melting point, making it easy to process (Du et al., 2022). The SLS parameter optimization of PCL scaffolds had been extensively and vigorously performed. Williams et al. (2005) varied the laser power from 1 to 7 W in steps of 1 W at a constant scan speed (3,810 mm/s) and powder bed temperature (40°C) to determine the suitable laser power. Partee et al. (2006) reported that the optimal SLS processing parameters of PCL powder can be optimized by systematic factorial experimental design so that the dimensional accuracy can reach within 3–8% of the design specification, and the density is about 94% of the full density. Doyle et al. (2015) studied the influence of the laser power and scanning spacing on the dimensional accuracy of PCL scaffolds. In the recent SLS process parameter optimization by Tortorici et al. (2021), not only the dimensional accuracy of PCL scaffolds but also the mechanical stability is considered. These studies provide good guidance for SLS preparation of PCL scaffolds, and their optimization parameters have been directly used in many cases (Sudarmadji et al., 2011; Bobbert et al., 2017). However, since the effects of these sintering variables are often interdependent, the optimization of the SLS process parameters of PCL scaffolds is still a challenge.

Therefore, in addition to the widely concerned mechanical properties and structural accuracy of scaffolds, the effects of the SLS process parameters on the surface properties of PCL scaffolds should be systematically studied. After all, the microgeometry and roughness of the scaffolds have a direct and significant impact on their bioactivity. Compared with the post-treatment surface modification method, it is expected that the regional SLS processing method under the optimized parameters achieves the one-step integration of bulk mechanical properties and surface properties, which will not only greatly save manufacturing time but also improve the stability of scaffold performance.

**FIGURE 1:**
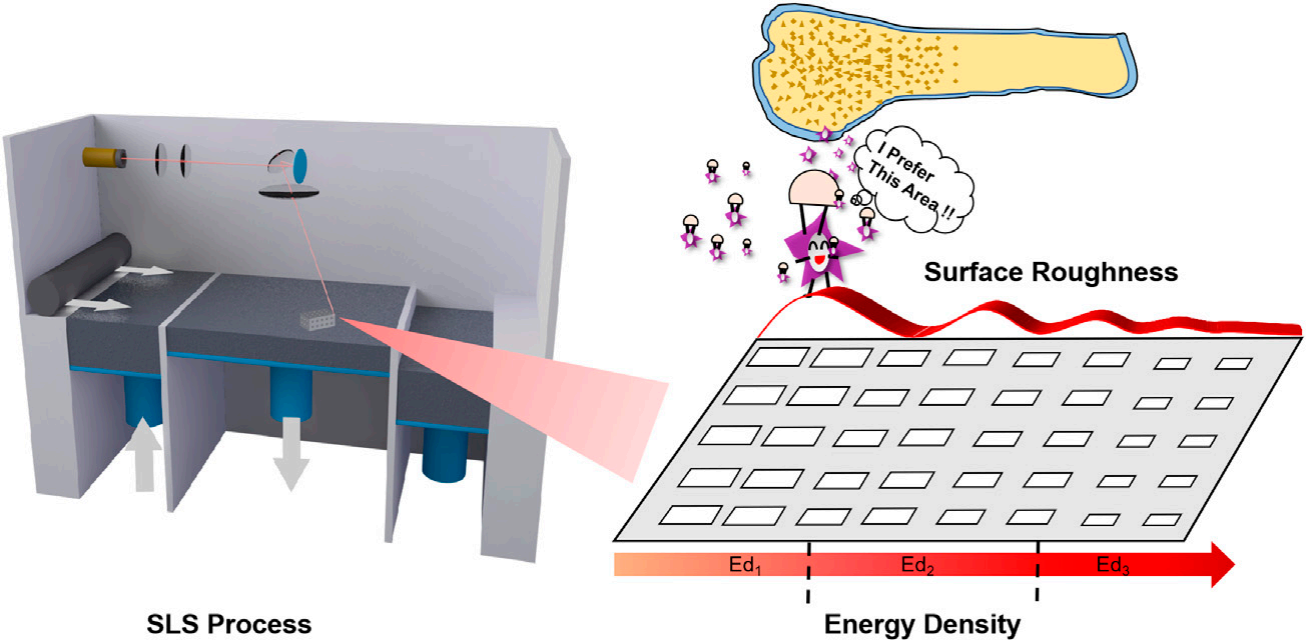
The Schematic diagram of this work.

In this study, we took the energy density model (EDM) as the theoretical basis for optimizing the SLS process parameters, which was originally proposed by Nelson (1993), and it has been used in the SLS parameters by many researchers (Gibson and Shi, 1997; Ho et al., 1999; Beal et al., 2009). The basic properties of PCL powder, including morphology and thermal behavior, were evaluated. Three SLS energy densities (Ed_1_, Ed_2_, and Ed_3_) were determined according to the energy density theory (Figure 1). Five PCL scaffolds with different laser powers and scanning speeds were prepared, and then their dimensional accuracy, mechanical strength, and surface properties were characterized. Finally, the bioactivities of PCL scaffolds prepared under different SLS parameters were compared through the attachment and proliferation of MC3T3-E1 cells on the scaffolds.

## 2 Materials and Methods

### 2.1 Characterization of Polycaprolactone Powder

Polycaprolactone powder (CAPA^®^ 6500 PCL) was purchased from Solvay (Belgium).

#### 2.1.1 Powder Morphology

The surface morphology of the PCL powder was characterized by using a scanning electron microscope (SEM, FEI, Quanta FEG 250, United States).

#### 2.1.2 Particle Size Distribution

The average particle size of the PCL powder was determined by using a Mastersizer (Malvern, Mastersizer 2000, United Kingdom).

#### 2.1.3 Thermodynamic Properties

The thermodynamic properties of the PCL powder are determined by using differential scanning calorimetry (DSC, TA, Q2000, United States) and thermogravimetric analysis (TGA, TA, Q5000, United States) with a heating rate of 10°C/min.

### 2.2 Design, Fabrication, and Characterizations of Porous Scaffolds

#### 2.2.1 Model Design

**FIGURE 2:**
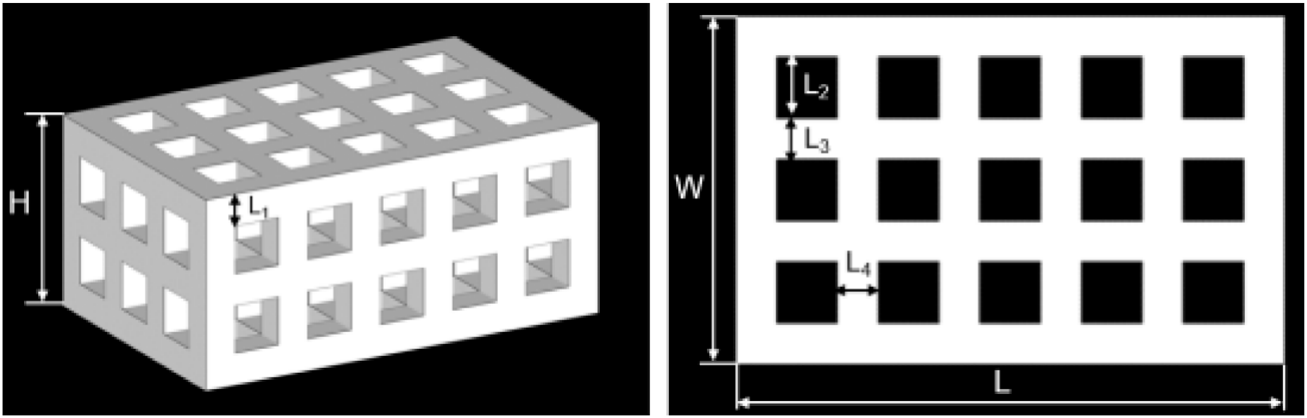
The 3D model of the porous scaffold for SLS.

A tetragonal porous scaffold with a three-dimensional (3D) orthogonal periodic porous square architecture was designed by Magics 21.0 (Materialise, Belgium), as shown in Figure 2. The dimensions and porosity of the model are listed in Table 1. The design was converted to STL file format.

**TABLE 1:** Parameters of the 3D porous scaffold model.

Parameters of the 3D porous scaffold model							
H (mm)	W (mm)	L (mm)	L_1_ (mm)	L_2_ (mm)	L_3_ (mm)	L_4_ (mm)	Porosity (%)
12.0	17.0	27.0	2.0	3.0	2.0	2.0	45.75

#### 2.2.2 Selective Laser Sintering Process of Scaffolds

An HK P320 SLS machine (Huazhong university of science and technology, China) with a controlled CO_2_ laser was used for scaffold manufacturing. After the scanning process, the scaffolds were held at the temperature for 2 h and then removed from the powder bed. Afterward, a high-power blower was used to blow away the unsintered powders on the surface and in the pores of the SLS-fabricated scaffolds from different angles as much as possible. Finally, all the scaffolds were rinsed with ddH_2_O several times until there were no obvious powders and debris. Then, the scaffolds were dried naturally for further use.

#### 2.2.3 Pore Size and Porosity

Micro-CT scanning (Skyscan, Bruker, Germany) was performed on five samples from each group. Meanwhile, the dimension and porosity were calculated from the 3D reconstructed images using CTAn1.13 software (Bruker, Germany).

#### 2.2.4 Water Absorption

The scaffolds were weighed after soaking in water for 30 min to get the wet weight. The water absorption of the scaffold was obtained by subtracting the dry weight from the wet weight and dividing it by the dry weight in the air, according to the following formula (Lei et al., 2012)

$$P=\left(W_{wet}-W_{dry}\right)/W_{dry}\times 100\%$$ (2-1)

*W*_wet_: wet weight of the scaffold; *W*_dry_: dry weight of the scaffold.

#### 2.2.5 Compressive Property

Compression tests of the scaffolds were performed using an RGM3000 electromechanical test frame (REGER, China) with 1 mm/min displacement rate. A total of five samples were tested for each type of scaffold. Their compression modulus was obtained from the initial region of the stress–strain curve.

#### 2.2.6 Scanning Electron Microscopy

The morphology of the scaffold’s surface and fracture surface were observed by scanning electron microscopy (SEM, FEI, Quanta FEG 250, United States).

#### 2.2.7 Surface Roughness

The surface roughnesses (SRs) from the top surface of circular specimens with different processing parameters were measured by using an Optical digital microscope (DSX510, Olympus, Japan). Sa and Sq are described as indicators. Sa is the arithmetic mean deviation, which is defined as the arithmetic average or centerline average from the centerline. Sq (root mean square value) represents the positive square root of the arithmetic mean of the value of the squares of the values in the set (Sachdeva et al., 2013).

### 2.3 *In Vitro* Biological Evaluation

#### 2.3.1 Protein Adsorption

The scaffolds with different SLS parameters were immersed overnight in a green fluorescent protein (GFP) solution that was expressed by *E. coli*. The scaffolds were taken out and carefully cleaned with phosphate buffer solution (PBS) three times to remove the unabsorbed GFP. Then, the scaffolds were observed under a fluorescence microscope (Leica OMI4000B, Germany) with the laser and camera parameters conditions consistent when taking fluorescent photos of the different groups.

#### 2.3.2 Cell Culture

MC3T3-E1 (mouse embryo osteoblast precursor cells, Chinese Academy of Medical Sciences, China) cells were cultured in Dulbecco’s modified Eagle’s medium (DMEM) containing 10% fetal bovine serum (FBS, Biochrom AG), 1% penicillin/streptomycin (P/S, Biochrom AG), and 1% L-glutamine (GlutaMAX, Invitrogen) and incubated at 37°C in a humidified atmosphere with 5% CO_2_. Cells were trypsinized (Gibco, United States) at approximately 80% confluency, and in passage 3 or 4, cells were used for seeding.

#### 2.3.3 Cell Seeding on the Scaffold

Five kinds PCL discs with the same diameter (5 mm) and height (1 mm) but different SLS parameters were used for *in vitro* experiments. At first, all the scaffolds were sterilized for 30 min by UV light and severally moved into the 24-well plate and marked. Each well was added 2 ml 1 × 10^3^ cells/ml cell suspension. Three repetitions were set for every group (Ed_1_, Ed_21_, Ed_22_, Ed_23_, and Ed_3_). The plate was gently shaken so that the cells were evenly distributed over the PCL discs’ surface. After 24 h culture, the scaffolds with cells adhered were taken out and gently cleaned with PBS three times. Then, the scaffolds were placed into a new 24-well plate, and 2 ml fresh medium was added to continue the culture.

#### 2.3.4 Fluorescence Staining

The time points selected to evaluate the live/dead test of cells on the scaffolds were 1, 3, and 5 days after seeding. At each time point, the surfaces of the five kinds of scaffolds (Ed_1_, Ed_21_, Ed_22_, Ed_23_, and Ed_3_) with cells were carefully cleaned three times with sterile PBS. The cells were incubated in a solution containing 2.5 μM calcein acetoxymethylester (Calcein/AM, 40747ES76, Yeason, China) and 4.5 μM propidium iodide (PI, 40747ES76, Yeason, China) diluted in PBS for 30 min at room temperature, and then the excess dye was rinsed by PBS. After that, taking out the sample and laying it on the rectangular cover glass sheet, the cell surface was placed on the bottom. Live (green) and dead (red) cells were identified by using a laser confocal machine (FV3000, Olympus, Tokyo, Japan).

#### 2.3.5 Cell Proliferation

The diluted suspensions (2 ml), containing different cell densities, were added into the wells of 24-well plates and cultured for 24 h. Specifically, the number of cells in each well was 0, 500, 1,000, 2,000, 5,000, 10,000, 20,000, and 40,000. Discarding the medium and cleaning three times with PBS, 450 µl medium and 50 µl CCK8 kit (BestBio, Shanghai, China) solution were added into the wells and then incubated at 37°C for another 2 h. Then, the culture medium was mixed thoroughly, and the supernatant with a volume of 100 µl was taken to a 96-well plate to measure the absorbance at 450 nm with a microplate reader (BL340, Biotech, United States). With the number of cells as the abscissa and the absorbance value as the ordinate, the standard curve was drawn and the relationship between the number of cells and the absorbance value was obtained by linear regression fitting. CCK8 test of the cells on the scaffolds was repeated every 24 h for 5 days. The number of cells on the scaffold with five different SLS parameters at each selected time point (days 1, 2, 3, 4, and 5 after cell seeding) was calculated according to the obtained equation.

#### 2.3.6 Statistical Analysis

The results of the experiments were statistically analyzed using the Origin software (version 2020, OriginLab, United States). All the data were presented as the mean ± SD. Statistical analyses among the multiple group data were carried out using a one-way analysis of variance (ANOVA) test to determine the significant differences. Tukey’s post-hoc test was used to determine the difference between any two groups with *p < 0.05 considered statistically significant.

## 3 Results

### 3.1 Selective Laser Sintering Theoretical Energy Density Model and Parameter Optimization of Polycaprolactone Powder

**FIGURE 3:**
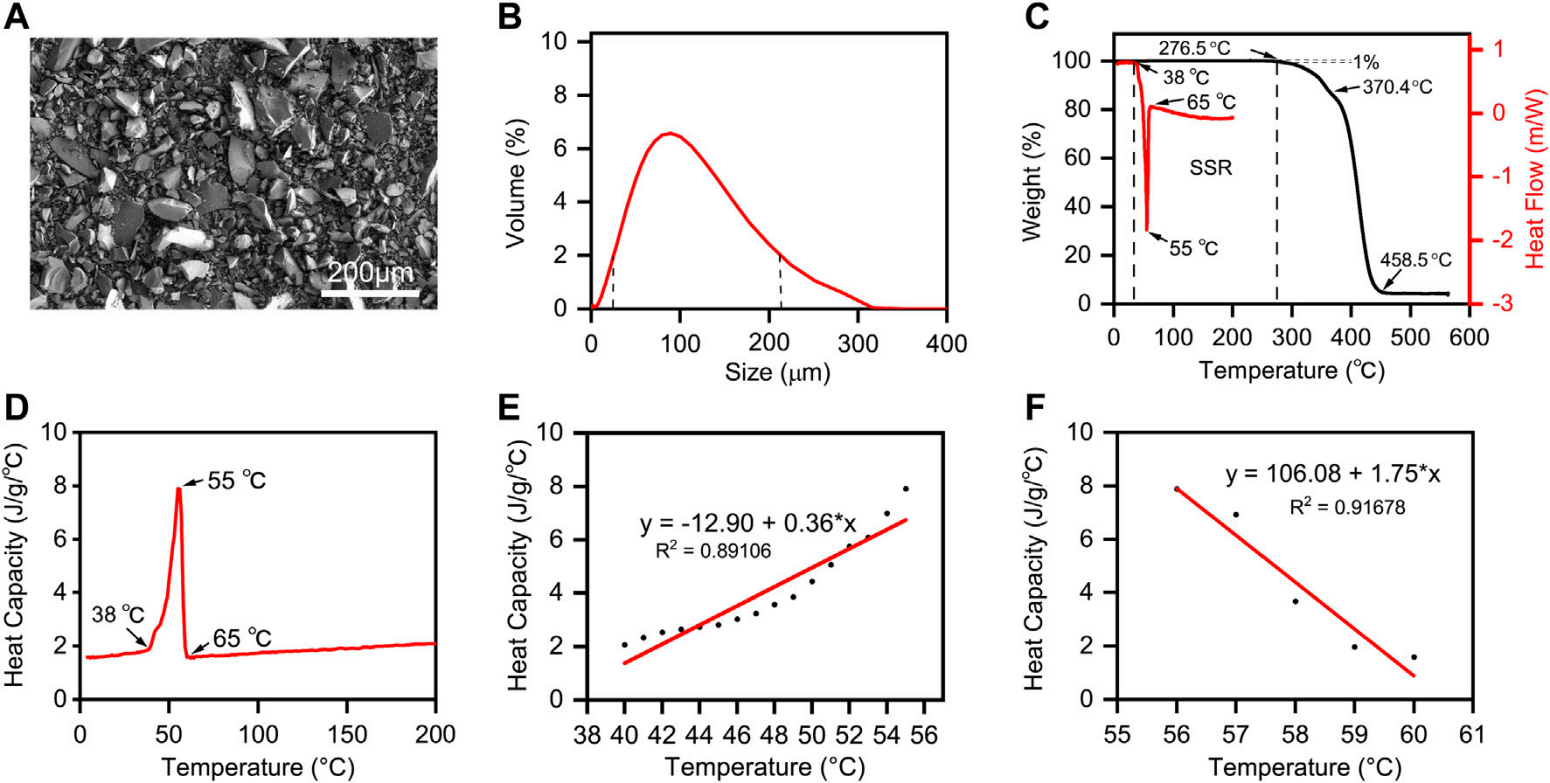
The Properties of PCL powder. **(A)** SEM image and **(B)** size distribution of PCL powders; **(C)** DSC and TGA analysis of PCL powder. The endo- and exothermal heat flow of PCL powders was characterized by heating and cooling from 0°C to 200°C at the rate of 10°C/min. The weight ratio of PCL decomposition started from 0°C to 600°C at the rate of 10°C/min. The stable sintering region (SSR) is indicated from the onset of melting to the onset (1%) of decomposition. **(D)** Plot of the temperature-dependent specific heat capacity of PCL powders from 0°C to 200°C. **(E)** Linear fitting of heat capacity with temperature from melting initiation to the melting point. **(F)** Linear fitting of heat capacity with temperature from the melting point to the recrystallization temperature.

The SEM image (Figure 3A) exhibited the uniformly nonspherical microscopic morphology of PCL powder. As shown in Figure 3B, the curve shows good symmetry. The PCL particle size distribution increased from 28 to 200 μm when the value of volume percent is greater than 2%, and the average particle size is about 90 μm. The size range of the PCL powder meets the requirements of SLS printing because the excessive size of the powder will affect the powder spreading process, and the too small size of the powder will cause the agglomeration of the powder (Shi et al., 2016). Hence, the minimum layer thickness was chosen as 0.15 mm.

According to the theoretical EDM, the offset melting temperature (*T*_mf_) and onset decomposing temperature (*T*_ds_) are the key points. We defined the temperature range from *T*_mf_ to *T*_ds_ as “Sintering stable range (SSR)” (Berretta et al., 2016). Differential scanning calorimetry (DSC) and thermogravimetric analysis (TGA) are applied to identify the SSR of PCL, and their curves and SSR region are shown in Figure 3C. We can conclude that the SSR of PCL is 38.0°C–276.5°C, and the sintering temperature should be within. Additionally, the heat capacity of PCL with different temperature ranges is shown in Figure 3D, while the linear fitting of heat capacity with temperature is shown in Figures 3E,F. *C*_PP_ means the heat capacity of PCL in the powder form, and *C*_Pm_ means the heat capacity of PCL in the liquid phase. To calculate the energy per volume required for melting *E*_mv_ and the energy per volume required for decomposing *E*_dv_ listed in Eqs 3-1, 3-2, the linear data fittings, applied for *C*_PP_ and *C*_Pm_ curves, the corresponding linear equations, and the other important material properties of PCL powders are listed in Table 2.

**TABLE 2:** Material properties of PCL powders.

Material properties	Value
Specific heat (*C*p*, J/g°C)	C = 0.0227e^0.1059^T, (30–65°C) C = 0.0034 T + 1.400, (65°C–277°C)
Melting temperature (*T*_m_, °C)	52.96
Onset melting temperature (*T*_ms_, °C)	38.0
Offset melting temperature (*T*_mf_, °C)	65.22
Onset decomposing temperature (*T*_ds_, °C)	276.5
Modified density (ρ∗, g/cm^3^)	1.1–7.81 × 10^−4^T + 0.519 × 10^−6^T^2^
Packing fraction (ϕ)	0.4
Light absorptivity (α, %) at 10.6 μm	0.9 Franco et al. (2010)

Based on the material properties of PCL powders, we used a theoretical method to investigate the influences of sintering parameters based on the thermal properties of PCL powders which were obtained from the above experiments. To sinter PCL powders effectively and prevent their decomposition, we should calculate *E*_mv_ and *E*_dv_ (Yuan et al., 2017). They are defined as

$$E_{m\nu }\mathrm{=\rho *}\left(T\right)\varphi \overset{T_{m}}{\underset{T_{b}}{\int }}C_{p}^{*}\left(T\right)dT,\;\left(T_{b}<T<T_{\mathrm{mf}}\right)$$ (3-1)

where *T*_b_ is the preheating temperature of laser sintering, *T*_mf_ is the onset and offset of melting, the PCL powder density ρ^∗^(*T*) is a function of temperature, and the modified specific heat *C*_p_^∗^is a function of temperature. φ is the packing factor of polymer powders.

$$E_{\mathrm{d\nu}}\mathrm{=\rho *}\left(T\right)\overset{T_{\mathrm{ds}}}{\underset{T_{\mathrm{mf}}}{\int }}C_{p}^{*}\left(T\right)\mathrm{dT,}\left(T_{b}<T<T_{\mathrm{mf}}\right)$$ (3-2)

where *T*_ds_ is the onset of decomposing, which is indicated by the 1% weight loss in the decomposition plot.

The energy density (*E*_d_) is a comprehensive numerical parameter to express the critical parameters of the SLS process including laser power (P), scanning speed (V), the layer thickness of each layer (H), and the hatch space (D)

$$E_{d}\mathrm{=P/VHD}$$ (3-3)

These energy calculations should satisfy a relationship expressed as

$$E_{m\nu }<\alpha E_{d}<E_{d\nu }$$ (3-4)

where *α* is the effective heat absorptivity of polymer powders during the SLS process.

As can be seen, the material properties of PCL powders are listed in Table 2. In addition, to ensure the minimum of the thermal gradient, the powder bed temperature must be maintained as close as possible to the onset melting point; otherwise, the powder bed would get caked. Usually, the powder bed temperature is maintained between 3 and 15°C below the onset melting point. Hence, we set the powder bed temperature as 35°C. According to the energy calculation formulas described above, the evaluations of *E*_mv_, *E*_dv_, and *E*_vol_ for PCL powders are listed in Table 3.

**TABLE 3:** Evaluations of *E*_mv_, *E*_dv_, and *E*_d_ of PCL powders.

Energy parameter	Value
Volume energy for melting (*E*_mv_, J/mm^3^)	0.090
Volume energy before decomposition (*E*_dv_, J/mm^3^)	0.184
Energy input range of laser (*E*_d_, J/mm^3^)	0.100–0.204

According to the energy input theoretical range of laser *E*_d_ (0.100–0.204 J/mm^3^), we chose three different densities Ed_1_, Ed_2_, and Ed_3_ by varying the laser power to 2, 4, and 6 W with the same scanning speed of 1,500 mm/s. Furthermore, to compare the actual effect of laser power and scanning speed on the properties of scaffolds, the laser power and scanning speed were varied simultaneously to keep the energy density constant, marked as Ed_21_, Ed_22_, and Ed_23_. The main process parameters are shown in Table 4.

**TABLE 4:** Main SLS process parameters of PCL scaffolds in this study.

Energy density	SLS process parameters			
Ed (J/mm^3^)	P (W)	V (mm/s)	D (mm)	H (mm)
Ed_1_ (0.089)	2	1,500	0.1	0.15
Ed_21_ (0.178)	2	750	0.1	0.15
Ed_22_ (0.178)	4	1,500	0.1	0.15
Ed_23_ (0.178)	6	2,250	0.1	0.15
Ed_3_ (0.267)	6	1,500	0.1	0.15

### 3.2 Characterizations of Polycaprolactone Scaffolds With Different Selective Laser Sintering Parameters

**FIGURE 4:**
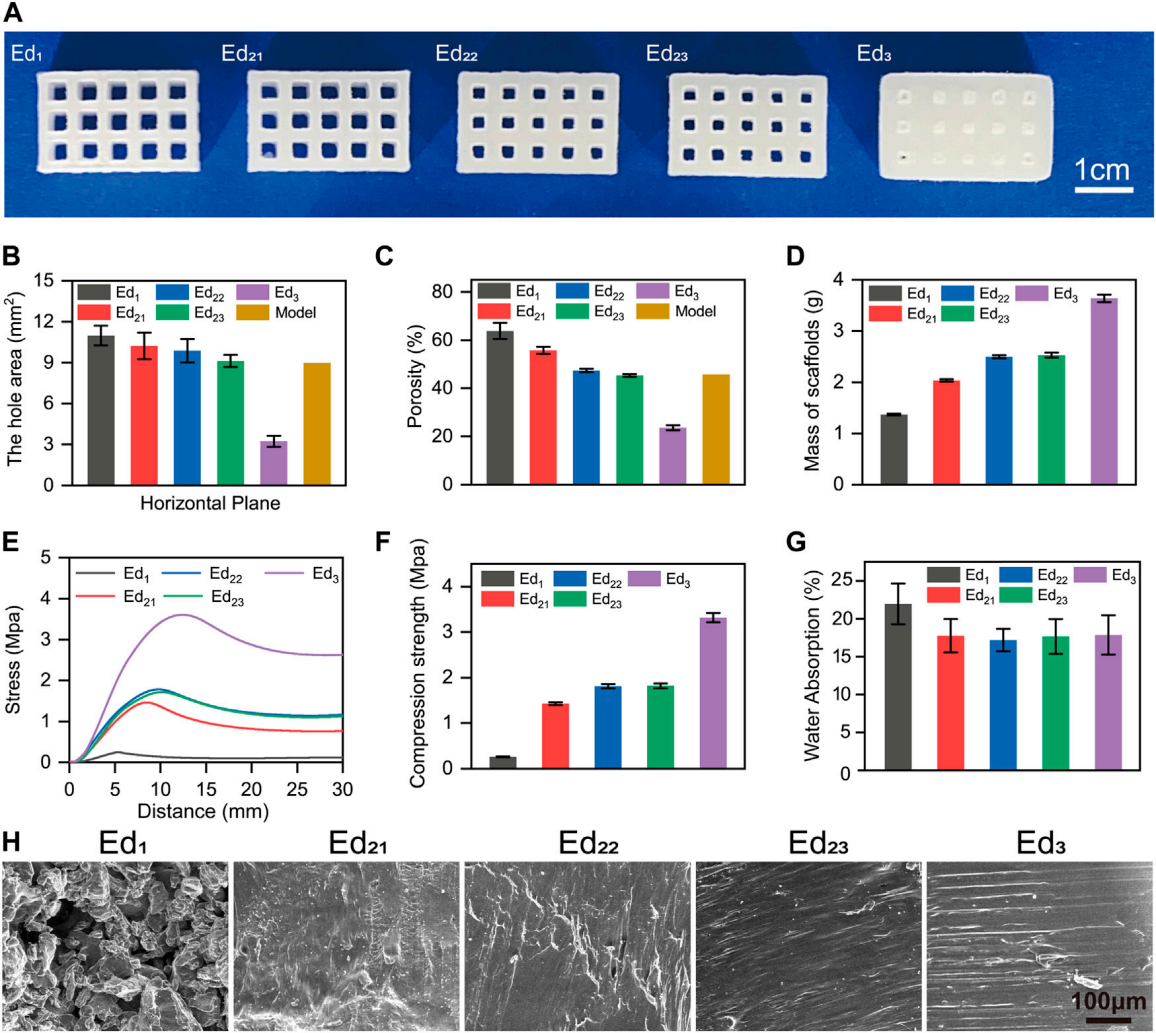
Characterizations of five kinds PCL scaffolds with different SLS parameters. **(A)** Photo of SLS-fabricated PCL scaffolds, **(B)** horizontal pore area, **(C)** porosity, **(D)** mass, **(E)** stress–strain curves, **(F)** maximum compressive strength, **(G)** water absorption, and **(H)** cross-section SEM images of scaffolds.

As shown in Figure 4A, five kinds PCL scaffolds (Ed_1_, Ed_21_, Ed_22_, Ed_23_, and Ed_3_) with different SLS parameters were successfully produced. Subsequently, the 3D reconstruction images of the scaffolds by micro-CT are shown in Supplementary Figure S1. It is easily noticed that their pore sizes were different. Therefore, the area of pores was measured from the horizontal direction (Figure 4B) and vertical direction (Supplementary Figure S2). Increasing the energy density by increasing the laser power from 2 W (Ed_1_) to 4 W (Ed_22_) and 6 W (Ed_3_) at a fixed scanning speed (1,500 mm/s) resulted in a decrease in the pore area. Due to the “bonus-z” effect (Ho and Gibson, 2000), the difference between vertical pores was more inapparent than that of horizontal pores. Specifically, in the horizontal direction, the measured pore area was 11.05 mm^2^ when the energy density was the lowest in this study at 0.089 J/mm^3^ (Ed_1_). The energy density increases to 0.178 J/mm^3^ (Ed_22_), and the pore area decreases to 9.88 mm^2^. Further increasing the energy density to 0.267 J/mm^3^ (Ed_3_), the originally designed 9 mm^2^ pore was only 3.24 mm^2^ with the lowest shape fidelity. When the laser power and scanning speed were increased in the same proportion under the same energy density (Ed_2_), the pore size was decreased from 10.26 mm^2^ (Ed_21_) to 9.17 mm^2^ (Ed_23_).

The porosity of scaffolds (Figure 4C) was consistent with the results of pore size. It can be observed that the porosity decreased from 63.80% to 23.60% when increasing the energy density from 0.089 (Ed_1_) to 0.267 (Ed_3_) J/mm^3^. The porosity was decreased from 55.78% (Ed_21_) to 45.31% (Ed_23_) as the laser power and scanning speed were increased in the same energy density (Ed_2_) as well.

Meanwhile, Figure 4D illustrates that the mass increased from 1.371 g (Ed_1_) to 3.638 g (Ed_3_) with the increase in energy density and the masses of scaffolds with the same energy density were 2.036 g (Ed_21_), 2.497 g (Ed_22_), and 2.530 g (Ed_23_).

The mechanical strength is also an important parameter for bone tissue scaffolds. Therefore, the compression strength and the stress–strain curves are assessed in Figures 4E,F. First, we found that all the stress–strain curves show the same tendency. Then, our results indicate that the improved mechanical properties can be achieved by the energy density increased from 0.089 to 0.267 J/mm^3^, and the mechanical performance of the scaffolds improved from 0.260 to 3.320 MPa. Moreover, when the energy density is the same (Ed_2_), the compression strength increased from 1.429 (Ed_21_) to 1.779 (Ed_22_) and 1.844 (Ed_23_) MPa with the increase of laser power and scanning speed.

As shown in Figure 4G, according to the study of Kim and Kim (2015) and our inference, the water absorption of the Ed_1_ group (21.94%) was much higher than that of other groups, which was related to the existence of a large number of unsintered PCL particles and undesigned micropores on the scaffolds.

The SEM images of the cross-section of the different scaffolds (Figure 4H) can explain the mechanical properties described above. As can be seen, the micropores disappeared and the cross-sectional microstructure became dense gradually with the increase in energy density. Meanwhile, the same tendency was also observed from Ed_21_ to Ed_23_.

**FIGURE 5:**
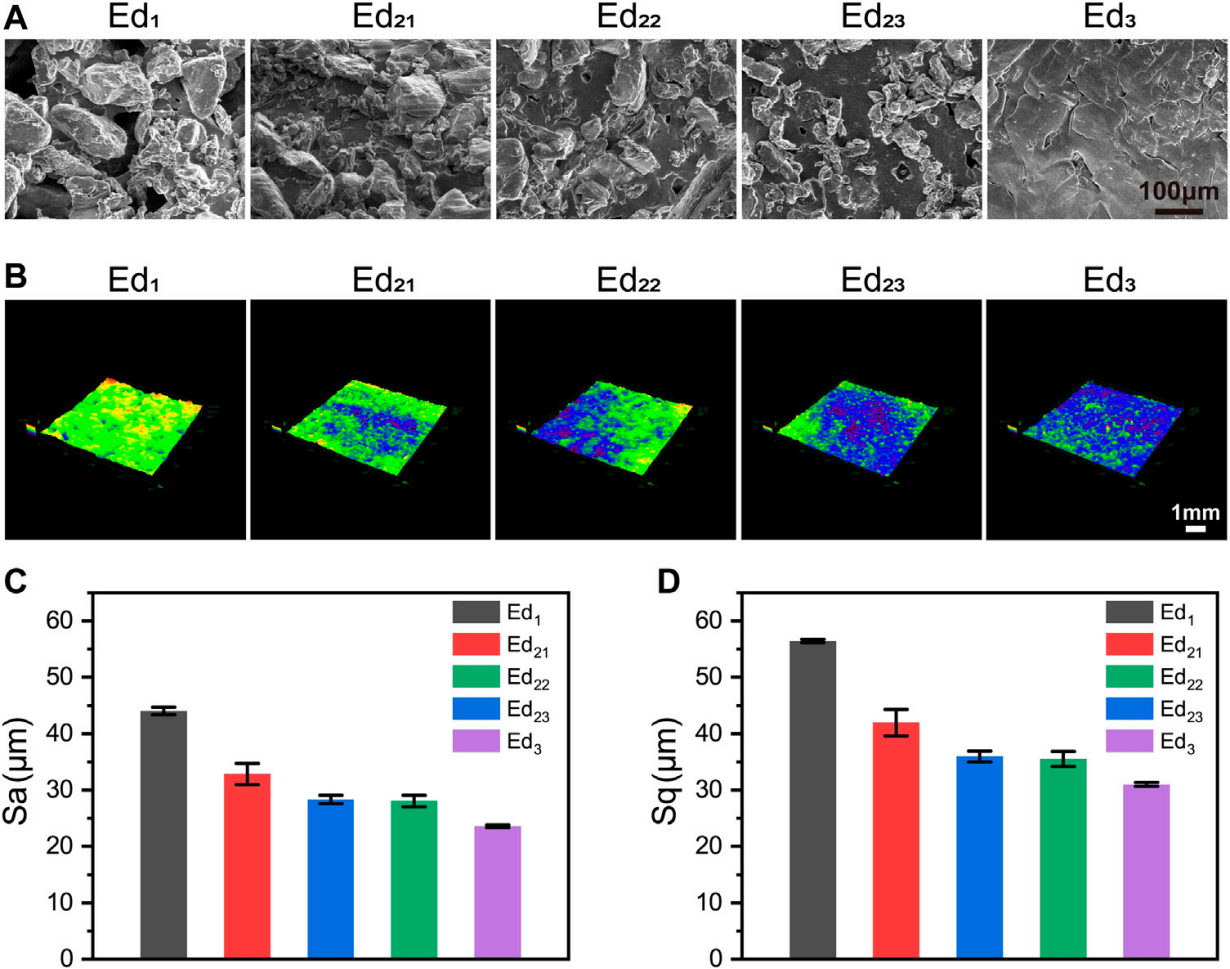
Characterizations of SR with different SLS parameters. **(A)** Surface SEM images of scaffolds; **(B)** 3D morphology; **(C)** Sa and **(D)** Sq statistical of the samples’ surface.

The SEM images of the different scaffolds’ surfaces are shown in Figure 5A. There were a lot of semisintered particles which remained on the surface of the Ed_1_ scaffold, which was caused by a low energy density. These incomplete sintered PCL particles and undesigned pores resulted in a very rough surface of the Ed_1_ group. When the energy density was increased to Ed_2_, PCL particles were further fused so that the surface morphology became smoother, but some peaks were still found protruding from the surface. Moreover, compared with the three different scaffolds in the same energy density (Ed_2_), with the increase in laser power and scanning speed, smaller unsintered PCL particles and fewer undesigned micropores were observed on the scaffolds’ surfaces. As the energy density was further increased to Ed_3_, the peaks and the microstructure pores disappeared so that the surface became very compact and smooth.

As shown in Figures 3D, 5B, topographical images were used to evaluate the SR. Meanwhile, the quantitative results of SR, Figures 5C,D (Sa) and (Sq), indicated that the Ed_1_ scaffolds showed highly roughened surfaces with average roughnesses of 56.42 μm (Sa) and 44.02 μm (Sq), and Ed_3_ scaffolds had the smoothest surface with average roughnesses of 31.02 μm (Sa) and 23.60 μm (Sq). These results indicated that the SR was decreased with increasing energy density. Similarly, the SR values of Ed_21_, Ed_22_, and Ed_23_ were measured: the average values of Sa were 32.84, 28.32, and 28.05 μm, respectively; the values of Sq were 41.95, 35.97, and 36.62 μm, respectively. Interestingly, the SR of the specimens with the same energy density was decreased with increasing laser power and scanning speed.

### 3.3 *In Vitro* Biocompatibility of Polycaprolactone Scaffolds With Different Selective Laser Sintering Parameters

**FIGURE 6:**
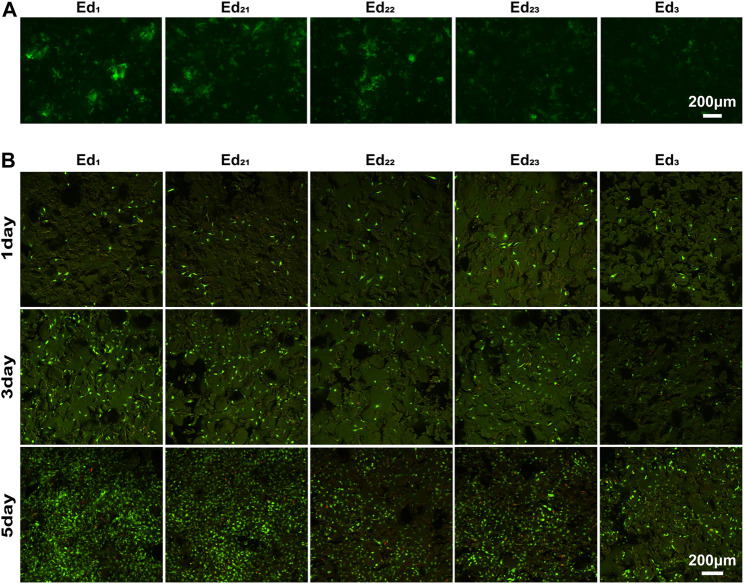
Qualitative analysis of *in vitro* biological properties of scaffolds. **(A)** Fluorescence images of GFP adsorption on the surface of scaffolds after 24 h. **(B)** Confocal laser images of MC3T3-E1 cells growing on the surface of scaffolds for 1, 3, and 5 days after live/death staining.

Generally, the composition of this adsorbed protein layer is a key mediator of cell behavior (Ngandu Mpoyi et al., 2016) and is very important for the biocompatibility of scaffolds. Therefore, Figure 6A shows the protein adsorption ability of the SLS-derived scaffolds when incubated with GFP for 24 h. Specifically, with the increase in energy density, fewer fluorescent areas appeared on the scaffolds when fluorescence images were taken under the same shooting parameters, inferring that less GFP was attached to the surface. Compared with the Ed_2_ and Ed_3_ groups, proteins seem to be more readily adsorbed to the surface of the Ed_1_ scaffold. Therefore, a lower energy density or lower laser power and scanning speed at the same energy density are favorable for protein adsorption on the scaffold surface. More protein on the surface will facilitate the cells to adhere on the scaffold, according to Kim et al. (2019).

The viability of MC3T3-E1 cells was assayed using live/dead staining after 1, 3, and 5 days of cell culture, where live cells were stained with calcein acetoxymethylester (Calcein-AM, green) and dead cells were stained with propidium iodide (PI, red) under a fluorescence microscope (Figure 6B). It is easily seen that all the scaffolds possessed the capability for cell proliferation, and the number and optical density of live cells on the scaffold surface increased with culture time prolonging. Very few dead cells were observed for all the culture time points, indicating that even after processing with SLS with a wide range of process parameters, PCL remains cytocompatible (Tortorici et al., 2021). After 1 day of culturing, the cells were evenly distributed on the scaffold and the number of live cells on all the scaffolds was similar. These phenomena demonstrated that the uniformly initial conditions of the cells on the scaffolds were suitable for further comparison of the biological differences on different scaffold surfaces.

Confocal laser scanning microscopy images displayed that the surfaces of all scaffolds were uneven with the cells spatially distributed. The cells could be observed at different levels within a range in the direction of observation, while the range of the Ed_1_ group is the largest. Moreover, the number of cells on scaffolds with the same energy density (Ed_21_, Ed_22_, and Ed_23_) showed a trend that increased with the laser power and scanning speed. Evidently, a large proportion of live cells adhered to the Ed_1_ and Ed_2_ scaffold’s surface where cells attached tightly with the well-flattened and well-spread morphology and grew in colonies without contact inhibition so that cells formed a confluent layer at day 5. However, cells adhering to the Ed_3_ group were sparsely distributed and presented a morphology with more round and less filopodia. Summarily, the distribution range, number, and viability of cells increased significantly with declining energy density.

**FIGURE 7:**
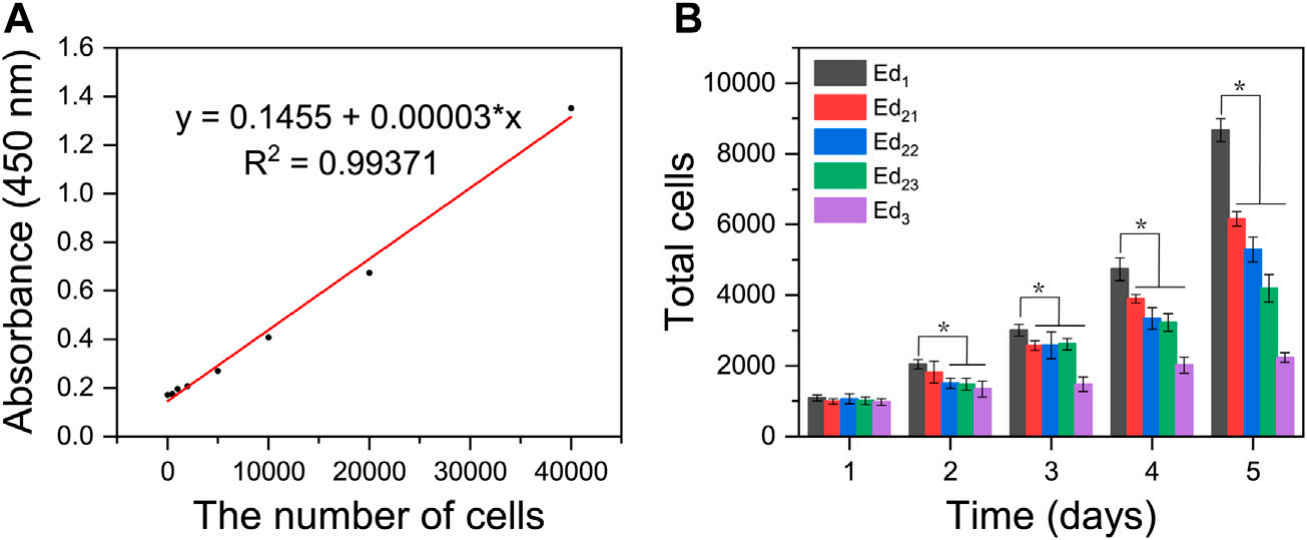
Quantitative analysis of *in vitro* biological characteristics of scaffolds. **(A)** Absorbance value at 450 nm of different amounts of MC3T3-E1 cells. **(B)** Proliferation activity of the adhered MC3T3-E1 cells on different scaffolds after 1, 2, 3, 4, and 5 days of incubation, detected by the CCK8 assay. (*n* = 7, error bar represents the mean ± SD, * indicates a significant difference compared with Ed_1_, *p* < 0.05).

A linear function was formed between the amounts of cells and the absorbance value at 450 nm based on Figure 7A. Through quantitative analysis, the same number of cells (1 × 10^3^) were initially inoculated on all the five types of scaffolds, and with the extension of time, the cells showed a tendency of continuous growth, manifesting that all the SLS-fabricated scaffolds supported MC3T3-E1 cell proliferation with low toxicity and high safety. However, it was easily seen that MC3T3-E1 cells showed different proliferation on the printed scaffolds with different parameters. The results were consistent with that of AM/PI staining. As shown in Figure 7B, the proliferation rates and maximum cell number on the Ed_1_ group were significantly higher than those of Ed_2_ and Ed_3_ groups when the culture time was increased to 5 days (*p* < 0.05) and also exhibited a negative correlation between energy density and cellular proliferation. In addition, the number of cells on the scaffolds with the same energy density (Ed_2_) also showed a significant difference that decreased as the laser power and scanning speed increased at day 5 (Ed_21_, Ed_22_, and Ed_23_; *p* < 0.05).

## 4 Discussion

In this study, the theoretical EDM was used for the first time to systematically optimize the SLS process parameters of PCL scaffolds. Five PCL scaffolds with different SLS processing parameters were manufactured successfully, which means that the calculated energy density range was pretty suitable based on PCL powder properties and EDM. According to our attempts not demonstrated in this study, PCL porous scaffolds were hard to form when the energy density was not in this range. In addition, as the energy density gradually increased, the performance of PCL scaffolds showed obvious differences in porosity, mechanical properties, surface properties, and cytocompatibility. Moreover, under the same energy density, the change of laser power and scanning speed in the same proportion was basically the same as the change in energy density. However, the effects of varying laser power and scanning speed respectively need to be further compared.

Most importantly, although none of the scaffolds prepared from a wide range of SLS process parameters displayed cytotoxicity, the cytocompatibility of scaffolds showed a negative correlation with the significantly varied energy density. All the *in vitro* experimental results indicate that the cell adhesion and proliferation effect of the Ed_1_ scaffold was superior to that of other groups, which was evidently related to the improved roughness of the scaffold’s surface that was caused by the decrease of energy density. The lower energy density prevents the PCL particles on the surface of the scaffold from melting completely, which leads to a lot of unsintered PCL particles adhered to the surface. Meanwhile, these unsintered PCL particles also resulted in poor packing of the particles in the powder bed so that the tendency of the layers to curl with the roller increases, which restricts the next layer from proper sintering, resulting in the rough surface. Previous studies have demonstrated that roughness might be responsible for improving cell responses such as proliferation (Van Bael et al., 2013). Similarly, it can be analyzed from our results that rough surfaces not only provide a wider growth space to accommodate more cells but also allow the scaffold to absorb more water and expose more protein adsorption sites (Figures 5D, 6A), thus affecting cell attachment and proliferation. The same phenomenon can be observed when the energy density was the same and the laser power and scanning speed were changed in the same proportion.

The increase in energy density will reduce the shape accuracy of the scaffold in terms of pore size and porosity. There might be two reasons: one is that the higher energy density leads to a higher heat-affected area, resulting in a larger shrinkage of consolidated PCL powder, and the other is that the higher energy density with a higher temperature gradient transfers heat from the center to the edge of the molten pool more effectively, generating a wider molten pool (Czelusniak and Amorim, 2020). Hulbert et al. (1971) claimed that a minimum pore size of 100 μm is necessary for the porous implant materials so that it is conducive to the growth of neovascular substances to transport nutrients needed for cell growth and the exchange of metabolites. Therefore, considering the overall biocompatibility of scaffolds, the scaffolds with low porosity prepared by using the Ed_3_ parameters are unsuitable for implantation. The *in vivo* biological properties of scaffolds with different porosities made by varying SLS parameters need to be further studied.

However, as observed in the SEM images (Figure 5A), the increase in energy density results in a coherent structure with good adhesion and low porosity, which improved compression strength in a step-like pattern from 0.26 to 3.32 MPa. The synergistic effect of incomplete sintering of PCL particles and large porosity leads to poor compression strength of the Ed_1_ scaffold. During the long process of bone tissue repair, the 3D structure of the scaffold without the necessary mechanical capacity may collapse so that it cannot occupy the expected space for cells’ growth.

This finding will bring some new enlightenment to the field of bone tissue scaffold manufacturing as well. First of all, it must be recognized that the change of the SLS process parameters for preparing PCL scaffolds will not only affect the porosity, mechanical properties, and surface properties of the scaffolds but also affect the biological properties. The excellent biological properties of bone tissue scaffolds are the original and desirable results of using SLS technology to fabricate the PCL scaffolds. Therefore, when the SLS process parameters of biomedical polymer materials such as PCL are optimized, the biological properties should become a key factor to determine whether the technical parameters are appropriate or even should be given top priority in the repair of non-load-bearing bone defects. In addition, it should be considered that different SLS technological parameters can be used in different regions to prepare tissue engineering scaffolds in the future. Specifically, a higher energy density should be applied when the main part of the inner scaffold is sintered, while a lower energy density should be used for printing scaffolds in areas where the surface is in direct contact with the cells or tissues, increasing the surface roughness and enabling the scaffolds to have good mechanical properties and biocompatibility. Evidently, compared with other surface modification methods, such as grit blasting, porous metallic coatings, and plasma spray coatings (Le Guéhennec et al., 2007; Park et al., 2016; Orinaková et al., 2020), continuous processing of the SLS not only greatly saves time but also ensures the stability and repeatability of the scaffold performance.

## 5 Conclusion

EDM can be used for narrowing the energy density range of PCL sintering and guiding the direction of SLS process parameter optimization. Within our study, changing the SLS process parameters affects not only the porosity and mechanical properties but also SR so that it affects the biological properties of the scaffolds. Increasing the energy density or the laser power and scanning speed at the same energy density will smooth the surface of the scaffold so that the biological performance of the scaffolds will be decreased. Biological properties should be an important factor to optimize the SLS process parameters of the PCL scaffolds for non-load-bearing bone repairing. In the future, SLS regional processing, like PCL, will be a necessity when making bone scaffolds with gradient performance so that further advantages of SLS will be presented.

## Data Availability

The raw data supporting the conclusion of this article will be made available by the authors, without undue reservation.
