# Supplementary material for: Surface Roughness and Biocompatibility of Polycaprolactone Bone Scaffolds: An Energy-Density-Guided Parameter Optimization for Selective Laser Sintering
Source: Front Bioeng Biotechnol. 2022 Jul 11;10:888267. doi: 10.3389/fbioe.2022.888267 (PMC9309791; doi:10.3389/fbioe.2022.888267)
Supplement: Supplementary file 1 [file DataSheet1.docx]

**Supplementary materials**


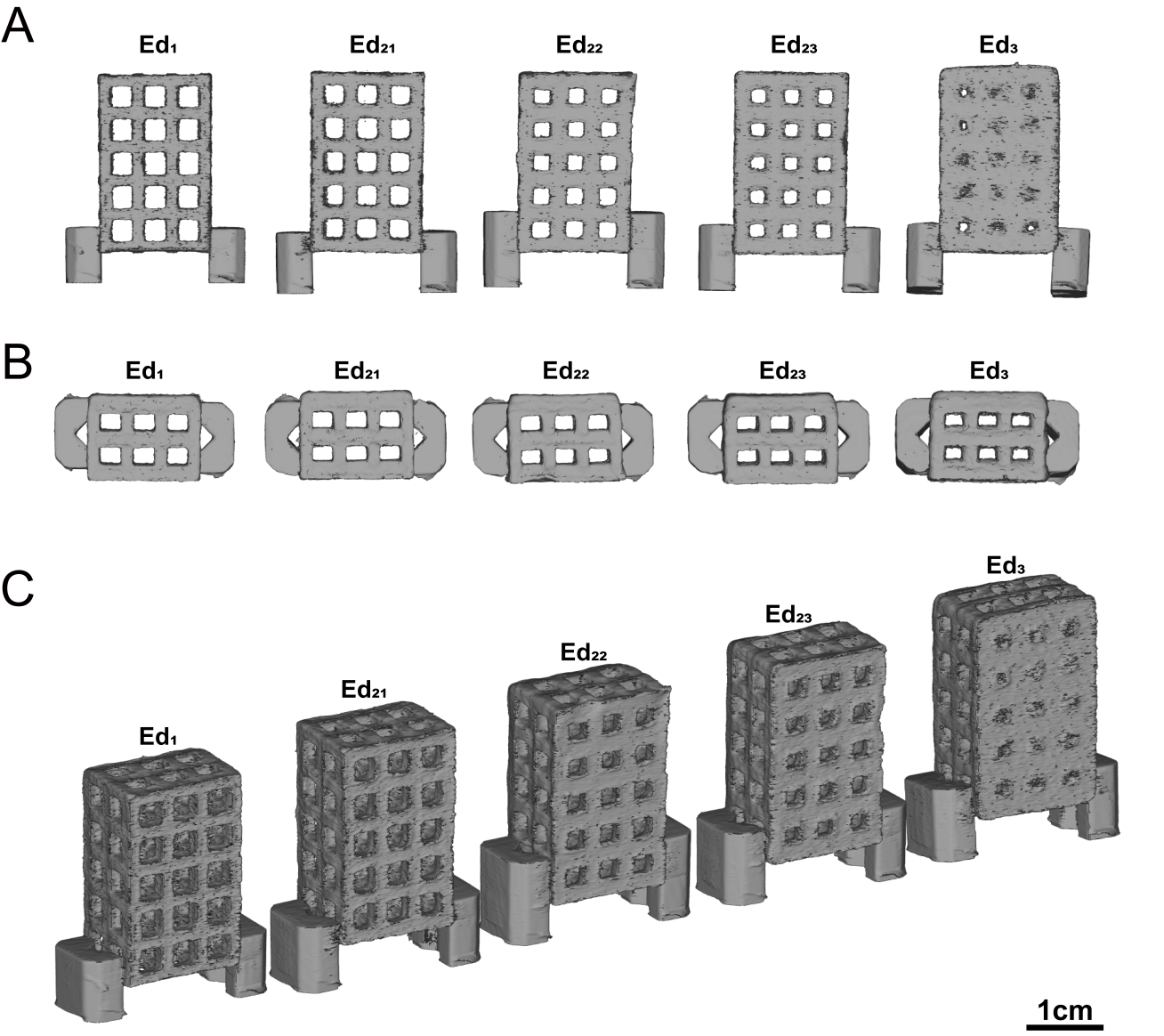
**Fig. S1.** 3D images in different directions of the scaffolds.

**
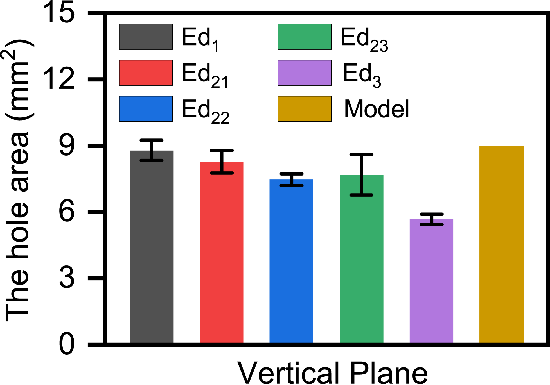
**

**Fig. S2.** The vertical pore area of scaffolds.
